# Characterising Cytokine Gene Expression Signatures in Patients with Severe Sepsis

**DOI:** 10.1155/2013/164246

**Published:** 2013-07-02

**Authors:** Robert Grealy, Mary White, Patrick Stordeur, Dermot Kelleher, Derek G. Doherty, Ross McManus, Thomas Ryan

**Affiliations:** ^1^Department of Clinical Medicine, Institute of Molecular Medicine, Trinity College Dublin, St James's Hospital, Dublin 8, Ireland; ^2^Service d'Immunologie, Hôpital Erasme, Brussels, Belgium; ^3^Department of Clinical Immunology, Institute of Molecular Medicine, Trinity College Dublin, St James's Hospital, Dublin 8, Ireland

## Abstract

*Introduction*. Severe sepsis in humans may be related to an underlying profound immune suppressive state. We investigated the link between gene expression of immune regulatory cytokines and the range of illness severity in patients with infection and severe sepsis. *Methods*. A prospective observational study included 54 ICU patients with severe sepsis, 53 patients with infection without organ failure, and 20 healthy controls. Gene expression in peripheral blood mononuclear cells (PBMC) was measured using real-time polymerase chain reaction. *Results*. Infection differed from health by decreased expression of the IL2, and IL23 and greater expression of IL10 and IL27. Severe sepsis differed from infection by having decreased IL7, IL23, IFN**γ**, and TNF**α** gene expression. An algorithm utilising mRNA copy number for TNF**α**, IFN**γ**, IL7, IL10, and IL23 accurately distinguished sepsis from severe sepsis with a receiver operator characteristic value of 0.88. Gene expression was similar with gram-positive and gram-negative infection and was similar following medical and surgical severe sepsis. Severity of organ failure was associated with serum IL6 protein levels but not with any index of cytokine gene expression in PBMCs. *Conclusions*. Immune regulatory cytokine gene expression in PBMC provides a robust method of modelling patients' response to infection.

## 1. Introduction

 Severe sepsis, an overwhelming inflammatory response to an underlying infection, remains a lethal disease that is present in over a third of European intensive care patients [1]. In previous studies this group reported a link between gene expression of specific immune regulatory cytokines in peripheral blood mononuclear cells (PBMC) and the presence of severe sepsis in patients with infection [2–5]. The specific cytokine groups whose differential expression were most closely associated with severe sepsis included the T-cell homeostatic cytokines IL2 and IL7 [6]; cytokines modulating the interaction between innate and adaptive immunity, namely, IL23 and IL27 [7]; and cytokines enhancing bactericidal activity, namely, TNF*α* and IFN*γ* [8]. In these preliminary studies differential gene expression of a range of other cytokines, such as TGFb-1 were weakly linked, or such as IL1*β*, IL4, IL12, and IL18 were not linked, with the occurrence of severe sepsis in patients [9].

In this new study, we sought to validate the findings of our initial studies into a single larger study in a new cohort of patients, integrating all of the cytokines most closely related to the occurrence of severe sepsis identified from prior individual studies. In addition we planned to recruit equal numbers of patients with infection and severe sepsis in order to more accurately characterise cytokine gene expression signatures specific to severe sepsis as opposed to infection. Lastly we derived an index of cytokine gene expression in order to test the strength of the association between cytokine gene expression and the presence of severe sepsis in patients with infection. 

There was a strong association between the presence of severe sepsis in response to infection and differential immune regulatory cytokine gene expression. 

## 2. Materials and Methods

 The study was conducted in St James's Hospital, Dublin, Ireland. Approval was obtained from St James's Hospital Ethics Committee and informed written consent was obtained from each patient or next of kin. Exclusion criteria included (a) malignancy, (b) chemotherapy, (c) infection with human immunodeficiency virus, (d) immunosuppressive therapy including long-term corticosteroids, and (e) immunological disease. Three patients groups were recruited.

Patients presented with severe sepsis as an admitting diagnosis to the intensive care unit were enrolled. Patients satisfied the criteria of (i) a documented bacterial infection by clinical and/or microbiological evidence and (ii) severe sepsis, with at least one severe sepsis-related organ failure. In patients with severe sepsis, blood sampling was performed upon admission to intensive care within 48 hours of meeting the inclusion criteria and/or 7 days after meeting the inclusion criteria. 

Severity of illness was characterized upon admission to ICU using the Simplified Acute Physiology Score (SAPS II) [10] and the sequential organ failure assessment (SOFA) scoring systems on day 1 and again on day 7 [11]. The source of infection necessitating the ICU admission and the occurrence of an ICU death or survival to ICU discharge were recorded. 

Hospital in-patients with clinical and bacteriological evidence of infection who did not develop organ failure were recruited. In order to avoid recruiting patients with trivial infection or patients with systemic illness misdiagnosed as infection, patients with infection and a confirmed bacteraemic episode but no organ failure were recruited. In the infection group, blood sampling was performed within 24 hours of the first reported positive blood culture authorised by a consultant microbiologist. 

Twenty healthy hospital staff and laboratory co-researchers were recruited and informed consent was obtained. Blood sampling from healthy controls was at one time point only.

Peripheral blood mononuclear cells were isolated by density centrifugation, lysed, and stored at −80° following isolation. Total RNA was extracted using the RNeasy kit (Qiagen, Crawley, West Sussex, UK). Absolute quantification of gene expression was determined using TaqMan Gene expression assays performed on an ABI Prism 7000 or ABI 7900 HT (Applied Biosystems). Interleukin-6 protein measurement was determined by ELISA (Quantikine, RnD Systems, Minneapolis, MN, USA). Immune phenotyping of peripheral mononuclear cells was performed on a Beckman Coulter Cyan ADP Cytometer. Further details of these methods are provided in Appendix A.

Groups were compared using Wilcoxon rank sum test and Kruskal-Wallis test, with Bonferroni correction for multiple comparisons. Spearman rank correlation coefficient was used to analyse the relation between continuous parameters. Gene expression in patient groups was compared by logistic regression analysis. Data analysis was performed using JMP 9.0 statistical package (SAS, Cary, NC, USA).

## 3. Results

**Figure 1:**
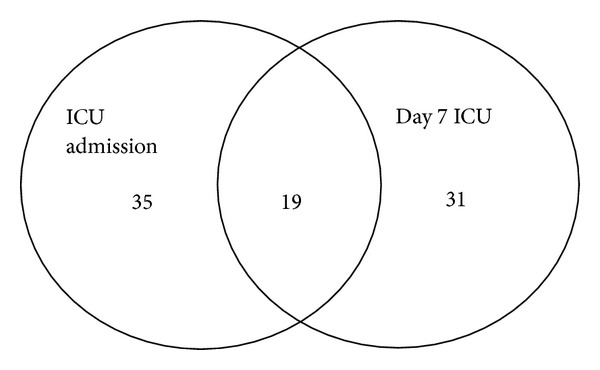
Number of patients with severe sepsis: blood was drawn for study at ICU admission, at day 7 of ICU admission, or both.

Patient demographics for the three patient groups are presented in Table 1. Twenty healthy controls were recruited. Fifty-three patients with infection and without organ failure were recruited. Fifty-four patients with severe sepsis had blood samples at ICU admission; 50 patients with severe sepsis had samples 7 days after ICU admission, with 19 of these severe sepsis patients having samples drawn at ICU admission and 7 days later (Figure 1). 

Immunophenotyping from patients with severe sepsis and healthy controls is detailed in Table 2. CD3^+^ CD127^+^ lymphocytes, a population of naïve and memory T cells, were decreased in septic patients.

Gene expression of cytokines in PBMC of healthy controls, patients with infection, and patients with severe sepsis is contained in Table 3. When healthy controls and patients with infection were compared, IL2 and IL23 gene expression was lower in patients with infection, and gene expression of IL10 and IL27 were greater in patients with infection. IL7, IFN*γ*, and TNF*α* gene expression was similar in these two groups, after correction for multiple comparisons. On multivariate regression, comparing cytokine gene expression in health and infection, IL10 (*P* = 0.02), IL23 (*P* = 0.01), IL27 (*P* = 0.01), and TNF*α* (*P* = 0.03) were significantly different. The area under a receiver operator curve for this regression model was 0.97. 

In patients with infection, cytokine gene expression was similar in patients with gram-positive and gram-negative infection. 

When patients with infection and patients with severe sepsis on ICU admission were compared, IL2, IL7, IL23, IFN*γ*, and TNF*α* gene expression was lower in patients with sepsis, while IL-27 gene expression was similar in these two groups (Table 3). Upon correction for multiple comparisons IL10 gene expression was greater in patients with severe sepsis. In a multivariate nominal logistic regression model comparing gene expression in patients with infection and those with sepsis at ICU admission, IL10 (*P* = 0.02), IFN*γ* (*P* < 0.0001), and TNF*α* (*P* = 0.03) retained statistical significance. The area under a receiver operator curve for this regression model was 0.88, with cut-off values of 3.1 for IL10, 2.5 for IFN*γ*, and 4.75 for TNF*α*.

At the time of ICU admission cytokine gene expression was similar in the severe sepsis patients with medical or surgical illness (Appendix B).

In the 19 septic patients with cytokine gene expression assayed on ICU admission and after 7 days of ICU stay, after Bonferroni correction for multiple comparisons, cytokine gene expression did not change over time (Appendix B). When gene expression for all patients with samples at the time of ICU admission was compared with all patients with gene expression data on the seventh day of ICU admission, cytokine gene expression was similar at ICU admission and 7 days later (Appendix B).

An index of cytokine gene expression was derived. This cytokine gene expression index consisted of the difference in log mRNA copy numbers for cytokines that were decreased in sepsis, namely, IFN*γ*, TNF*α*, IL7, and IL23, and the cytokine increased in sepsis, namely, IL10 (IFN*γ* + TNF*α* + IL7 + IL23–IL10). IL2 was excluded from this score as it was not assayed in all patients. 

**Figure 2:**
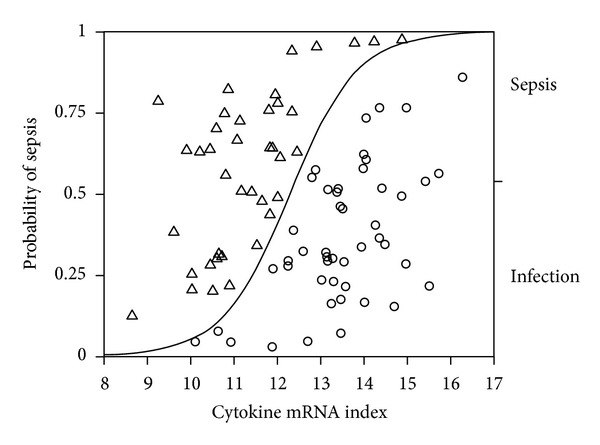
Probability of presence of sepsis in relation to cytokine mRNA index. Logistic regression analysis; model; *n* = 87, *r* ^2^ = 0.39, *P* < 0.0001.

This index was significantly different in healthy controls (median 14.2, interquartile range 13.6–15.3, *n* = 18), patients with infection (median 13.5, interquartile range 12.9–14.4, *n* = 47), and patients with severe sepsis at ICU admission (median 11.2, interquartile range 10.6–12, *n* = 40, *P* < 0.0001). When this index of cytokine gene expression in patients with severe sepsis at ICU admission and infection were included in a logistic regression analysis there was a significant relation between cytokine index and patient group (Figure 2; logistic regression model; *n* = 87, *r* ^2^ = 0.39, *P* < 0.0001); the odds ratio for developing severe sepsis increased by 3.6 per unit change of the score, and by an odds ratio of 18340 over the range of the score, with an area under the receiver operator characteristic curve of 0.887. This algorithm, at a cut-off value of 12.5, correctly identified 36 of 40 patients with sepsis and 39 of 47 patients with infection but without severe sepsis on day 1, giving a sensitivity of 90%, a specificity of 83%, a positive predictive value of 81%, and a negative predictive value of 90%.

In patients with severe sepsis there was no association between the severity of organ failure and cytokine gene expression or gene expression score in PBMCs. There was no association between mortality and PBMC cytokine gene expression. Serum IL6 levels were greater in patients with severe sepsis at ICU admission (median 741.8 pg/mL, IQR 578–1247, *n* = 50) than patients with infection (median 80 pg/mL, IQR 61–105, *n* = 49, *P* < 0.0001). In patients with severe sepsis there was a significant association between sequential organ failure assessment (SOFA) scores and serum IL6 protein levels at ICU admission and 7 days after admission (admission *P* < 0.001, Spearman *ρ* = 0.796, *n* = 49; day 7 *P* < 0.001, Spearman *ρ* = 0.812, *n* = 30).

When blood levels of IL-6 were included with the cytokine gene expression score in a multivariate analysis (logistic regression model; *n* = 87, *r* ^2^ = 0.76, *P* < 0.0001), both IL-6 (*P* = 0.001) and cytokine index (*P* = 0.02) retained statistical significance, and the area under a receiver operator curve was 0.98. 

## 4. Discussion

This study outlines a model of host response to infection based upon gene expression of immune regulatory cytokines in PBMCs, rather than soluble mediators of systemic inflammation. This data emphasises the central role of immune response in patients both with infection and severe sepsis, with a persistent abnormality of immune response in patients with severe sepsis. We propose a practical technique to quantify this immune response. 

There is a physiologic basis for the cytokines in this model, identified from prior pilot studies by this group, given that these cytokines regulate the immune response to infection. Both IL2 and IL7 regulate T-cell homeostasis, with IL2 produced in an autocrine manner upon T-cell activation [6]. IL7, produced by antigen-presenting cells induces naïve and memory T cells to differentiate into effector T cells. In this study it was notable that decreased IL7 gene expression characterised severe sepsis rather than infection. Both IL23 and IL27 are produced by antigen-presenting cells and regulate the interaction between innate and adaptive immunity. Specifically, IL23 acts primarily upon memory T cells, inducing differentiation to an effector phenotype [12]. In contrast IL27, with multiple potentially antagonist actions, induces T-cell IL10 production [13]. 

IFN*γ* and TNF*α* production by PBMCs is of pivotal importance in generating an appropriate bactericidal response to infection [8]. IFN*γ* induces HLA-DR expression by antigen-presenting cells, and this may be an important mechanism by which it reduces mortality in sepsis [14–16]. In this study it was notable that decreased IFN*γ* gene expression characterised severe sepsis rather than infection. IL10, a prototypic anti-inflammatory cytokine, is produced by a wide range of leukocytes in response to both infectious and noninfectious stimuli [17]. IL10 has significant immune suppressant effects [18–20]. 

CD3^+^ CD127^+^ lymphocytes, a population consisting of naïve and memory T cells, were decreased in patients with severe sepsis. CD127 is the IL7*α* receptor and is expressed by naïve and memory T cells: IL7 mediates expansion of naïve and memory T cells by binding to CD127. As cell surface expression of CD127 decreases after binding with IL7, thereby limiting the effect of IL7, effector T cells are CD127^−^. Thus the decrease in IFN*γ* in patients with severe sepsis was not associated with any alteration in effector or memory T cells. Immune suppression in sepsis and a decrease in inducible lymphocyte IFN*γ* gene production may alternatively be related to T-cell apoptosis, expression of inhibitory signalling molecules, and the exaggerated effects of regulatory T cells or a decrease in T-cell repertoire [21–25]. 

The pattern of cytokine gene expression in patients with severe sepsis is not reactive, as patients with infection exhibited similar pattern of dysregulated gene expression. Furthermore in thoracic surgery patients, a similar pattern of perioperative change in cytokine gene expression was observed to precede the onset of infection at a time when markers of systemic inflammation remained unchanged [4, 5]. Lastly innate familial patterns of leukocyte TNF*α* and IL10 production have been linked with risk for mortality in meningococcal disease [26]. 

The cytokines analysed in this study are immune regulators and effectors rather than mediators of systemic inflammation, which accounts for the apparent lack of association between severity of organ failure and cytokine gene expression. 

In the preliminary screening studies by this group, only blood levels of IL6 correlated with severity of organ failure, and hence IL6 was assayed as an index of inflammation [9]. In this and other studies, the severity of multiple organ failure was related to serum IL6 levels [27, 28]. IL6 is produced predominantly by hepatocytes and endothelial cells rather than peripheral blood lymphocytes and plays a pivotal role generating the organ damage of systemic inflammation [29–34]. While Goldie et al. reported that IL6 levels are greater in septic patients who succumb to their illness, the current study was too small to examine this association [35]. 

Studies examining serum levels of cytokines in patients with severe sepsis have shown inconsistent results [35–38]. Furthermore, serum cytokines emanate from many cell groups and thus reflect a global rather than cell-specific host response. In contrast, gene expression assays quantifying cellular mRNA expression from peripheral blood leukocytes allow sensitive and specific measurement of the functional state of specific immune cells and have already shown promise in the differentiation of patients at high and low risk for severe sepsis in the postoperative period [39]. Absolute quantitative RT-PCR or qPCR utilises internal standards of known concentrations with each PCR run enables faithful comparison of results of gene expression assays between patients [40, 41]. 

Microarray studies of leukocyte gene expression in septic patients have identified anomalous expression of immune-related genes in septic patients, without any discernable transition from proinflammatory to counter inflammatory response [42]. These studies examined gene expression in signalling pathways rather than effector cytokines, often in heterogenous cell lines from whole blood samples, using microarray technology rather than RT-PCR, and thus did not observe the same robust link between sepsis and cytokine gene expression. 

## 5. Conclusion

We characterised immune responses in patients with severe sepsis using molecular profiling with standard molecular biological techniques. Severe sepsis and infection in humans are strongly associated with an abnormal immune response. Severe sepsis in humans should be considered to represent a disorder of both immunity and inflammation. This approach may be important in future studies of severe sepsis.

## Figures and Tables

**Table 1:** Demographics of patients recruited to study. Values expressed as means and 95% confidence intervals.

	Severe sepsis (ICU)		Infection	Control
	ICU admission	Day 7 ICU		
*N*	54	32	53	20
Median age (yrs)	71 (60–76)	72 (61–79)	71 (49–81)	30 (27–33)
Gender				
Male	23 (54%)	16 (53%)	33 (62%)	8 (40%)
Female	27 (46%)	14 (47%)	20 (38%)	12 (30%)
Patient type				
Surgery	23	20	—	—
Medical	31	12	—	—
Bacteraemia				
Gram positive	10	—	24	—
Gram negative	3	—	29	—
APACHE II	21 (19–22)	18.4 (15.7–21.7)	—	—
SOFA	12 (9.1–15)	8.9 (6.9–10.7)	—	—
SAPS II	52 (47–57)	50 (42–58)	—	—
Septic shock	42 (84%)	15 (50%)	—	—
Respiratory infection	23 (46%)	6 (20%)	—	—
Abdominal infection	14 (28%)	16 (53%)	—	—
Other infection	13 (26%)	8 (27%)	—	—

SOFA: sequential organ failure assessment score; SAPS II: simplified acute physiology score; APACHE: acute physiology and chronic health evaluation.

**Table 2:** Lymphocyte subsets in patients with severe sepsis and healthy controls. All values are median and interquartile range. Analysis is by Wilcoxon rank sum test.

	Controls	Sepsis	*P*
*N*	6	6	
T lymphocytes			
CD3^+^ CCR7^+^ CD45RA^−^ (%)	6 (1.3–12.9)	4.2 (2.7–10)	ns
CD3^+^ CCR7low CD45RA^−^ (%)	23.5 (20–31.3)	21 (17–28.8)	ns
CD3^+^ CCR7^+^ CD45RA^+^ (%)	12.7 (4–24.6)	7.6 (5.3–17)	ns
CD3^+^ CCR7^−^ CD45RA^+^ (%)	28.2 (6.6–43)	18.1 (14.1–24)	ns
CD3^+^ CD127^+^ (%)	55 (53–59)	37 (26–43.6)	0.004
CD3^+^ CD127^−^ (%)	17.5 (14.2–21)	18.7 (7.1–24.7)	ns
CD16^+^ CD56^+^ (%)	8.3 (4.9–12.3)	1.9 (1–14)	ns
Monocytes			
CD14^+^	3.1 (2.8–3.2)	1.5 (1.3–2.9)	ns
CD16^+^	1.6 (0.8–3.2)	0.6 (0.4–1.2)	0.06
CD14^+^/16^+^	0.1 (0.1–0.2)	0.2 (0.1–1)	ns

**Table 3:** Cytokine mRNA levels in patients with severe sepsis and controls. Values denote copy numbers expressed as log_10_ per 10^7^ copy numbers *β*-actin. Values are expressed as medians and interquartile ranges. Analysis by Wilcoxon rank sum test between healthy controls, patients with mild sepsis and severe sepsis day 1.

Cytokine	Control	Control versus infection	Infection	Infection versus sepsis at ICU admission	Severe sepsis ICU admission
INF*γ*	3	ns	2.8	*P* < 0.0001	2.3
	2.7–3.3		2.6–3.2		1.8–2.6
	*n* = 20		*n* = 51		*n* = 53

TNF*α*	4.5	*P* = 0.04	4.9	*P* = 0.0008	4.5
	4.3–4.9		4.5–5.5		4.3–5
	*n* = 20		*n* = 48		*n* = 50

IL2	2.8	*P* = 0.0002	2.3	*P* = 0.002	2
	2.5–3.1		2.2–2.6		1.6–2.2
	*n* = 20		*n* = 50		*n* = 32

IL7	3.8	ns	3.7	*P* = 0.0004	3.4
	3.7–4.1		3.5–4		3.2–3.7
	*n* = 19		*n* = 52		*n* = 40

IL10	2.6	*P* < 0.0001	2.9	*P* = 0.003	3.2
	2.4–2.7		2.8–3.2		2.9–3.5
	*n* = 19		*n* = 52		*n* = 52

IL23	5.1	*P* = 0.0008	4.8	*P* < 0.0001	4.5
	5.0–5.4		4.6–5.1		4.3–4.8
	*n* = 20		*n* = 53		*n* = 51

IL27	2.5	*P* < 0.0001	3	ns	3.1
	2.3–2.7		2.8–3.3		2.7–3.4
	*n* = 19		*n* = 52		*n* = 50

## Appendices

### A. Methods

#### A.1. Real Time PCR

Approximately 20 mL of blood was collected into potassium ethylenediaminetetraacetic (K3EDTA) tubes (Greiner Bio-One, Gloucestershire, UK). Blood was stored at room temperature for not less than 2 hours prior to PBMC isolation. Whole anticoagulated blood was granulocyte depleted by dextran sedimentation. Following this step, buffy coat isolation was performed using density gradient centrifugation. Supernatant from the dextran step was aspirated and diluted 1 : 1 with phosphate buffered saline (Sigma). 25 mL of the buffered supernatant was carefully layered over 15 mL of Ficoll (Lymphoprep, Axis-Shield, Dundee, Scotland) in a 50 mL conical tube (Sarstedt). Tubes were centrifuged at 400 ×g for 23 minutes with brake off. The cellular interface layer between the serum and Ficoll containing the mononuclear cell fraction was carefully aspirated. Cells were washed twice with phosphate buffered saline (PBS) (Sigma) and lysed prior to storage using a kit lysis buffer (RLT buffer, Qiagen, West Sussex, UK). Lysates were stored at −80° until required.

Primer/probes for TNF*α*, IL2, IL7, IL10, and IL23 were obtained as precustomised mixes from Applied Biosystems (ABI, Warrington, UK). Custom primers and probes for INF*γ*, IL27, and *β*-actin were designed using the publicly available sequences and synthesised by ABI. Standards of known concentration of plasmid incorporating the target sequence for the qPCR assay were gifted by P. Stordeur or cloned de novo and validated using the PCR assay for the target of interest.

Total RNA was extracted using the RNEasy Mini kit, (Qiagen, West Sussex, UK). Genomic DNA was eliminated by on column DNAse digestion followed by elution of the RNA in RNAse-free water. RNA quality and concentration were determined spectrophotometrically (Nanodrop 8000, Thermo Scientific, DE, USA). cDNA for each sample was synthesised from 500 ng total RNA using a mastermix prepared per reaction as follows: first strand reaction buffer (5X) 6 *μ*L, dithiothreitol (DTT) 0.1 M 3 *μ*L, murine Moloney lymphotropic virus (mMLV) reverse transcriptase 1.25 *μ*L (all Invitrogen no. 28028-013), dimethyl sulfoxide 4.5 *μ*L (Sigma), deoxynucleotide triphosphates (dNTPs) (Promega no. U1420), RNase inhibitor (RNasin no. N2511, Promega), and random hexamers 2 *μ*L (Invitrogen). The final reaction volume was made up to 30 *μ*L with RNase-free water and incubated at 37°C for 1 hour on a thermal cycler.

Absolute quantification of gene expression was determined using TaqMan Gene expression assays performed on an ABI Prism 7000 or ABI 7900 HT (Applied Biosystems). Samples were run in duplicate or triplicate. Levels of gene expression were determined by reference to standard curves of known concentrations of copy numbers of the sequence of interest in addition to a reference gene (*β*-actin) performed with each run. Results were expressed as copy numbers of target mRNA per 10^7^ *β*-actin mRNA copy numbers.

#### A.2. Interleukin 6

Blood was also collected in a clot activating serum tube and centrifuged at 1200 rpm for 10 minutes at room temperature. Serum supernatant was aspirated and stored in microcentrifuge tubes at −80°C. First thaw samples were used for protein determination. Interleukin-6 protein measurement was determined by ELISA (Quantikine, RnD Systems, Minneapolis, MN, USA). The volume of serum for the ELISA was 100 uL done on a standard 96 well plate.

#### A.3. Flow Cytometry

The following fluorochrome-labelled antibodies, CD3 PeCF594, CCR7 PeCy7, CD45RA V450 (BD Biosciences), CD14VioGreen (Miltenyi Biotec, Surrey, UK), CD16 eFluor450, CD127 eFluor780 (eBiosciences, Hatfield, UK), and CD56FITC (Immunotools, Friesoythe, Germany), were added to 12 × 75 mm polypropylene tubes. 100 uL whole blood was added and the mixture incubated at room temperature in the dark for 15 minutes. Samples were then lysed (FACS Lyse, BD Biosciences) and washed with phosphate buffered saline supplemented with 1% bovine serum albumin and 0.01% sodium azide. Cells were subsequently fixed in 500 *μ*L of 1% paraformaldehyde (Sigma-Aldrich) in PBS solution. Analysis was performed on a Beckman Coulter Cyan ADP Cytometer. Spectral overlap compensation was performed using single colour fluorochrome controls combined with anti-immunoglobulin compensation particles or negative control (BD CompBeads, BD Biosciences). T cells, NK cells, and monocytes were defined as CD3^+^, CD16^+^ CD56^+^, and CD14^+^/CD16^+^ populations, respectively. Memory T cells were further defined as CD45RA^+^, central versus effector subtype, respectively, as CCR7^−^ or CCR7^+^, and differentiated versus naïve/memory, respectively, as CD127^−^ or CD127^+^. Results are expressed as % positive of gated population.

### B. Additional Data

See Supplementary Tables  4, 5, and 6 available online at http://dx.doi.org/10.1155/2013/164246.

## Abbreviations

ICU: Intensive care unit
PBL: Peripheral blood leukocyte
IFN*γ*: Interferon gamma
TNF*α*: Tumour necrosis factor alpha
IL: Interleukin
mRNA: Messenger RNA
mMLV: Murine moloney lymphotrophic virus
cDNA: Complimentary DNA
qRT-PCR: Quantitate real time polymerase chain reaction
ELISA: Enzyme-linked immunosorbent assay
ROC: Receiver operating characteristic.
